# Supplementary material for: Bayesian sample size determination using commensurate priors to leverage preexperimental data
Source: Biometrics. 2022 Mar 28;79(2):669–83. doi: 10.1111/biom.13649 (PMC10952893; doi:10.1111/biom.13649)
Supplement: Supplementary file 1 — Web Appendices A–E referenced in Section 2, Figures S2– S4 in Sections 3 and 4, and Appendices H–L for additional simulations, extended application to time‐to‐event data, and a brief user‐guide to apply the proposed methodology, are available at the Biometrics website on Wiley Online Library. Programming code for the sample size formulae and reproducing the numerical results, is posted online along with this paper, as well as available at GitHub: https://github.com/haiyanzheng/SSDcmspriors [file BIOM-79-669-s001.pdf]

Supporting Information for:
Bayesian sample size determination using commensurate priors to leverage pre-experimental data

by Haiyan Zheng, Thomas Jaki, James M. S. Wason

A. DERIVATION OF THE UNIMODAL *t* MIXTURE DISTRIBUTION

Given the joint distribution of $\tilde{\theta}_k$ and ν_k as a mixture of Normal-Gamma distributions, it follows that

$$\begin{aligned} f(\tilde{\theta}_k \mid \theta_k) &= \int f(\tilde{\theta}_k, \nu_k \mid \theta_k) \mathrm{d}\nu_k = \int f(\tilde{\theta}_k \mid \theta_k, \nu_k) g(\nu_k) \mathrm{d}\nu_k \\ &= w_k \frac{b_{01}^{a_{01}}}{\sqrt{2\pi}\Gamma(a_{01})} \int_0^\infty \nu_k^{a_{01}-\frac{1}{2}} \exp\left(-\frac{2b_{01}\nu_k + \nu_k(\tilde{\theta}_k - \theta_k)^2}{2}\right) \mathrm{d}\nu_k + \\ &\quad (1-w_k) \frac{b_{02}^{a_{02}}}{\sqrt{2\pi}\Gamma(a_{02})} \int_0^\infty \nu_k^{a_{02}-\frac{1}{2}} \exp\left(-\frac{2b_{02}\nu_k + \nu_k(\tilde{\theta}_k - \theta_k)^2}{2}\right) \mathrm{d}\nu_k \\ &= w_k \frac{b_{01}^{a_{01}}}{\sqrt{2\pi}\Gamma(a_{01})} \int_0^\infty \nu_k^{a_{01}-\frac{1}{2}} \exp\left(-\frac{\nu_k((\tilde{\theta}_k - \theta_k)^2 + 2b_{01})}{2}\right) \mathrm{d}\nu_k + \\ &\quad (1-w_k) \frac{b_{02}^{a_{02}}}{\sqrt{2\pi}\Gamma(a_{02})} \int_0^\infty \nu_k^{a_{02}-\frac{1}{2}} \exp\left(-\frac{\nu_k((\tilde{\theta}_k - \theta_k)^2 + 2b_{02})}{2}\right) \mathrm{d}\nu_k. \end{aligned} \tag{S1}$$

Let $z_A = \frac{\nu_k}{2} \cdot A$, where $A = (\tilde{\theta}_k - \theta_k)^2 + 2b_{01}$, and let $z_B = \frac{\nu_k}{2} \cdot B$, where $B = (\tilde{\theta}_k - \theta_k)^2 + 2b_{02}$. We can re-write (S1) as follows.

$$\begin{aligned} f(\tilde{\theta}_k \mid \theta_k) &= \int f(\tilde{\theta}_k, \nu_k \mid \theta_k) \mathrm{d}\nu_k \\ &= w_k \frac{b_{01}^{a_{01}}}{\sqrt{2\pi}\Gamma(a_{01})} \left(\frac{2}{A}\right)^{a_{01}+\frac{1}{2}} \int z_A^{a_{01}-\frac{1}{2}} \exp(-z_A) \mathrm{d}z_A + \\ &\quad (1-w_k) \frac{b_{02}^{a_{02}}}{\sqrt{2\pi}\Gamma(a_{02})} \left(\frac{2}{B}\right)^{a_{02}+\frac{1}{2}} \int z_B^{a_{02}-\frac{1}{2}} \exp(-z_B) \mathrm{d}z_B, \end{aligned} \tag{S2}$$

where $\int z_A^{a_{01}-\frac{1}{2}} \exp(-z_A) \mathrm{d}z_A$ and $\int z_B^{a_{02}-\frac{1}{2}} \exp(-z_B) \mathrm{d}z_B$ are Gamma intergrals. Therefore,

$$\begin{aligned} f(\tilde{\theta}_k \mid \theta_k) &\propto w_k b_{01}^{a_{01}} \left(\frac{2}{A}\right)^{a_{01}+\frac{1}{2}} + (1-w_k) b_{02}^{a_{02}} \left(\frac{2}{B}\right)^{a_{02}+\frac{1}{2}} \\ &\propto w_k \left(\frac{1}{b_{01}}\right)^{-a_{01}} \left(\frac{A}{2}\right)^{-a_{01}-\frac{1}{2}} + (1-w_k) \left(\frac{1}{b_{02}}\right)^{-a_{02}} \left(\frac{B}{2}\right)^{-a_{02}-\frac{1}{2}} \\ &\propto w_k \left(\frac{1}{b_{01}}\right)^{-a_{01}-\frac{1}{2}} \left(\frac{(\tilde{\theta}_k - \theta_k)^2 + 2b_{01}}{2}\right)^{-a_{01}-\frac{1}{2}} + \\ &\quad (1-w_k) \left(\frac{1}{b_{02}}\right)^{-a_{02}-\frac{1}{2}} \left(\frac{(\tilde{\theta}_k - \theta_k)^2 + 2b_{02}}{2}\right)^{-a_{02}-\frac{1}{2}} \\ &\propto w_k \left(\frac{(\tilde{\theta}_k - \theta_k)^2}{2b_{01}} + 1\right)^{-\frac{2a_{01}+1}{2}} + (1-w_k) \left(\frac{(\tilde{\theta}_k - \theta_k)^2}{2b_{02}} + 1\right)^{-\frac{2a_{02}+1}{2}}, \end{aligned} \tag{S3}$$

which demonstrates that the predictive prior distribution for each $\tilde{\theta}_k$, given a historical parameter θ_k, is a mixture of non-standardised t distributions. In particular, the location parameters are *identical* to θ_k, while the scale parameters are $\frac{b_{01}}{a_{01}}$ and $\frac{b_{02}}{a_{02}}$, respectively. In other words, the two mixture components correspond to $\frac{\tilde{\theta}_k-\theta_k}{\sqrt{b_{01}/a_{01}}}$ and $\frac{\tilde{\theta}_k-\theta_k}{\sqrt{b_{02}/a_{02}}}$ that follow classic Student's t distributions with $2a_{01}$ and $2a_{02}$ degrees of freedom, respectively.

B. MOMENTS AND OVERALL VARIANCE OF THE t MIXTURE DISTRIBUTION

For a finite mixture distribution denoted by $f(x) = \sum_i w_i h_i(x)$, its ℓ-th moment about zero exists and follows that

$$\mu^{(\ell)} = \mathbb{E}_f(x^{(\ell)}) = \sum_i w_i \mathbb{E}_{h_i}(x^{(\ell)}) = \sum_i w_i \mu_i^{(\ell)}, \tag{S4}$$

where $\mu_i^{(\ell)}$ is each ℓ-th moment of the mixture component $h_i(x)$. In our context, the constant prior mixture weights $w_1 = w_k$ and $w_2 = 1 - w_k$; while the components $h_i(\tilde{\theta}_k|\theta_k)$, $i = 1, 2$, are the non-standardised Student's t distributions, both centred at θ_k. We then know that $\mu_1^{(1)} = \mu_2^{(1)} = \theta_k$. We further constrain that $a_{01}, a_{02} > 1$ so that variances, denoted by σ_i^2, of $h_i(\tilde{\theta}_k|\theta_k)$ exist, as $\frac{b_{01}}{a_{01}-1}$ and $\frac{b_{02}}{a_{02}-1}$, respectively. Following Equation (S4), the overall variance for the mixture distribution can be computed as

$$\begin{aligned}
\mathrm{Var}(\tilde{\theta}_k|\theta_k) &= \mu^{(2)} - [\mu^{(1)}]^2 \\
&= \sum_i w_i \mu_i^{(2)} - \left[\sum_i w_i \mu_i^{(1)}\right]^2 \\
&= \sum_i w_i (\sigma_i^2 + [\mu_i^{(1)}]^2) - \left[\sum_i w_i \mu_i^{(1)}\right]^2 \\
&= \sum_i w_i \sigma_i^2 + \sum_i w_i [\mu_i^{(1)}]^2 - \left[\sum_i w_i \mu_i^{(1)}\right]^2 \\
&= w_k \frac{b_{01}}{a_{01}} \cdot \frac{2a_{01}}{2a_{01}-2} + (1-w_k)\frac{b_{02}}{a_{02}} \cdot \frac{2a_{02}}{2a_{02}-2} + w_k\theta_k^2 + (1-w_k)\theta_k^2 - [w_k\theta_k + (1-w_k)\theta_k]^2 \\
&= \frac{w_k b_{01}}{a_{01}-1} + \frac{(1-w_k)b_{02}}{a_{02}-1}.
\end{aligned}$$

C. ON THE GOODNESS OF THE NORMAL APPROXIMATION

As illustrated, $\tilde{\theta}_k$ follows a non-standard t mixture distribution that has its two components both centred at θ_k. We approximate it by a normal distribution $N\left(\theta_k, \frac{w_k b_{01}}{a_{01}-1} + \frac{(1-w_k)b_{02}}{a_{02}-1}\right)$, of which the variance was set equal to the overall variance of the unimodal t mixture prior.

This approximation is considered to be appropriate for the comparable coverage probabilities of a given credible interval, defined as $(\theta_k - r,\ \theta_k + r)$. Precisely, with the unimodal t mixture

distribution, the coverage probability can be given by

$$\begin{aligned}\mathbb{P}_t(|\tilde{\theta}_k-\theta_k|\le r) &= w_k \times \int_{\theta_k-r}^{\theta_k+r} h_1(\tilde{\theta}_k)\mathrm{d}\tilde{\theta}_k + (1-w_k)\times \int_{\theta_k-r}^{\theta_k+r} h_2(\tilde{\theta}_k)\mathrm{d}\tilde{\theta}_k \\ &= \frac{w_k\Gamma(\frac{2a_{01}+1}{2})}{\Gamma(a_{01})\sqrt{2\pi b_{01}}}\int_{-r}^{r}\left(1+\frac{(\tilde{\theta}_k-\theta_k)^2}{2b_{01}}\right)^{-\frac{2a_{01}+1}{2}}\mathrm{d}(\tilde{\theta}_k-\theta_k)+ \\ &\quad \frac{(1-w_k)\Gamma(\frac{2a_{02}+1}{2})}{\Gamma(a_{02})\sqrt{2\pi b_{02}}}\int_{-r}^{r}\left(1+\frac{(\tilde{\theta}_k-\theta_k)^2}{2b_{02}}\right)^{-\frac{2a_{02}+1}{2}}\mathrm{d}(\tilde{\theta}_k-\theta_k).\end{aligned} \tag{S5}$$

The coverage probability of $(\theta_k - r,\ \theta_k + r)$ given the normal approximation has a closed-form expression, and we further let it be of size $1-\alpha$:

$$\mathbb{P}_G(|\tilde{\theta}_k-\theta_k|\le r) = \Phi\left(\frac{r}{\sqrt{\frac{w_k b_{01}}{a_{01}-1}+\frac{(1-w_k)b_{02}}{a_{02}-1}}}\right) = 1-\alpha.$$

We may then solve this by giving $r = z_{\alpha/2}\sqrt{\frac{w_k b_{01}}{a_{01}-1}+\frac{(1-w_k)b_{02}}{a_{02}-1}}$, where $z_{\alpha/2}$ denotes the upper $(\alpha/2)$-th quantile of the standard normal distribution, i.e., $\Phi^{-1}(1-\alpha/2)$. Substituting this to (S5), we can derive the exact coverage probability of the interval in the non-standardised t mixture distribution, which is approximately $1-\alpha$.

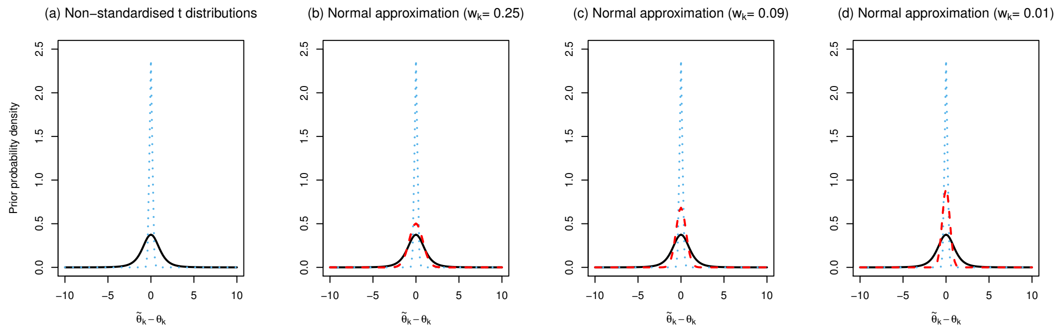

Figure S1: The t mixture distribution for $\tilde{\theta}_k - \theta_k$, with the components depicted using black solid and blue dotted curves. Its normal approximations, given by $\tilde{\theta}_k - \theta_k \sim N\left(0, \frac{w_k b_{01}}{a_{01}-1}+\frac{(1-w_k)b_{02}}{a_{02}-1}\right)$, are visualised using the red dashed curves in subfigures (b) – (d), under various choices of w_k.

For illustration, we specify that $\nu_k \sim w_k$Gamma(2, 2) + $(1-w_k)$Gamma(18, 3). Thus, $\tilde{\theta}_k - \theta_k$ follows a non-standardised t mixture distribution centered at 0, which may be approximated by a $N\left(0, \frac{w_k b_{01}}{a_{01}-1}+\frac{(1-w_k)b_{02}}{a_{02}-1}\right)$. Figure S1 displays some example density curves for $\tilde{\theta}_k - \theta_k$, with the component t distributions visualised in each subfigure. In subfigures (b) – (d), we overlie the density curve of the normal approximation, given prior mixture weights $w_k = 0.25, 0.09, 0.01$. As we observe, a smaller value of w_k tends to distort the approximated normal density curve further towards the more informative component t distribution.

Based on the normal approximation and setting $w_k = 0.25, 0.09, 0.01$, a 95% credible interval being symmetric about θ_k can be yielded by r = 1.559, 1.144, 0.865, respectively. Substituting these values of r to (S5), we compute the exact coverage probabilities based on the original t mixture distribution as 95.1%, 96.4%, and 95.5%, which are all close to 95% as expected. Finally, we note that the component distribution Gamma(2, 2) is quite extreme; with larger a_{01} and b_{01}, the approximation tends to be even better.

D. PREDICTIVE PRIOR THAT LEVERAGES MULTI-SOURCE PRE-EXPERIMENTAL DATA

We start from the normal approximation to the non-standardised t mixture distribution:

$$\tilde{\theta}_k \mid \theta_k \dot{\sim} N\left(\theta_k, \frac{w_k b_{01}}{a_{01}-1} + \frac{(1-w_k)b_{02}}{a_{02}-1}\right),$$

where θ_k has a (posterior) distribution $\theta_k|\boldsymbol{y}_k \sim N(m_k, s_k^2)$, or this distribution is characterised through elicitation of expert prior opinion on the difference in means. We then write

$$\tilde{\theta}_k = \theta_k + e,$$

where, independently to θ_k, we know $e \sim N\left(0, \frac{w_k b_{01}}{a_{01}-1} + \frac{(1-w_k)b_{02}}{a_{02}-1}\right)$. Now $\tilde{\theta}_k$ is just the sum of two independent normal random variables, which is fully determined by mean

$$\lambda_k = \mathbb{E}(\tilde{\theta}_k) = \mathbb{E}(\theta_k) + \mathbb{E}(e) = m_k,$$

and variance

$$\xi_k^2 = \mathrm{Var}(\tilde{\theta}_k) = \mathrm{Var}(\theta_k) + \mathrm{Var}(e) = s_k^2 + \frac{w_k b_{01}}{a_{01}-1} + \frac{(1-w_k)b_{02}}{a_{02}-1}.$$

E. POSTERIOR FOR THE DIFFERENCE IN MEANS WHEN THE VARIANCE IS UNKNOWN

We suppose that the unknown mean μ_Δ has a normal prior distribution specified based on K pre-experimental datasets, $N(\sum p_k\lambda_k, \sum p_k^2\xi_k^2)$, and the unknown variance σ_0^2 has an Inv-Gamma$(\frac{c}{2}, \frac{c\sum p_k^2\xi_k^2}{2})$ prior distribution. The quantity $c\sum p_k^2\xi_k^2/\sigma_0^2$ then has a $\chi^2(c)$ distribution. The posterior for μ_Δ can thus be given by

$$\begin{aligned}
f_p(\mu_\Delta \mid \boldsymbol{y}_1,\dots,\boldsymbol{y}_K,\boldsymbol{y}_{K+1}) &= \int \pi_p(\mu_\Delta,\sigma_0^2 \mid \boldsymbol{y}_1,\dots,\boldsymbol{y}_K,\boldsymbol{y}_{K+1}) g(\sigma_0^2)\mathrm{d}\sigma_0^2 \\
&\propto \int \mathcal{L}(\bar{x}_\Delta \mid \mu_\Delta,\sigma_0^2)\pi(\mu_\Delta \mid \boldsymbol{y}_1,\dots,\boldsymbol{y}_K) g(\sigma_0^2)\mathrm{d}\sigma_0^2 \\
&\propto \int \frac{1}{\sigma_0}\exp\left(-\frac{(\bar{x}_\Delta-\mu_\Delta)^2}{2\sigma_0^2\left(\frac{1}{n_A}+\frac{1}{n_B}\right)}\right)\cdot\exp\left(-\frac{(\mu_\Delta-\sum p_k\lambda_k)^2}{2\sum p_k^2\xi_k^2}\right)\cdot\frac{1}{(\sigma_0^2)^{\frac{c}{2}+1}}\exp\left(-\frac{c\sum p_k^2\xi_k^2}{2\sigma_0^2}\right)\mathrm{d}\sigma_0^2 \\
&\propto \exp\left(-\frac{(\mu_\Delta-\sum p_k\lambda_k)^2}{2\sum p_k^2\xi_k^2}\right)\int\frac{1}{(\sigma_0^2)^{\frac{c+1}{2}+1}}\exp\left(-\frac{1}{2\sigma_0^2}\left[\frac{(\bar{x}_\Delta-\mu_\Delta)^2}{\frac{1}{n_A}+\frac{1}{n_B}}+c\sum p_k^2\xi_k^2\right]\right)\mathrm{d}\sigma_0^2 \\
&\propto \exp\left(-\frac{(\mu_\Delta-\sum p_k\lambda_k)^2}{2\sum p_k^2\xi_k^2}\right)\left[\frac{(\bar{x}_\Delta-\mu_\Delta)^2}{\frac{1}{n_A}+\frac{1}{n_B}}+c\sum p_k^2\xi_k^2\right]^{-\frac{c+1}{2}}.
\end{aligned} \tag{S6}$$

The last step in (S6) follows, since the integrant is the kernel of the density for an Inv-Chi-square distribution with c degrees of freedom, multiplied by $\left[\frac{(\bar{x}_\Delta-\mu_\Delta)^2}{\frac{1}{n_A}+\frac{1}{n_B}}+c\sum p_k^2\xi_k^2\right]$. We then divide both terms in the square brackets by $c\sum p_k^2\xi_k^2$ and obtain

$$f_p(\mu_\Delta \mid \boldsymbol{y}_1,\dots,\boldsymbol{y}_K,\boldsymbol{y}_{K+1}) \propto \exp\left(-\frac{(\mu_\Delta-\sum p_k\lambda_k)^2}{2\sum p_k^2\xi_k^2}\right)\left[1+\frac{(\mu_\Delta-\bar{x}_\Delta)^2}{c\left(\frac{1}{n_A}+\frac{1}{n_B}\right)\sum p_k^2\xi_k^2}\right]^{-\frac{c+1}{2}}, \tag{S7}$$

which features the product of kernels of normal and non-standardised t distributions, with the latter of c degrees of freedom, shifted by $\bar{x}_\Delta$ and scaled by $\left(\frac{1}{n_A}+\frac{1}{n_B}\right)\sum p_k^2\xi_k^2$. Therefore, the marginal posterior relies on the distribution of $\bar{x}_\Delta$.

We can easily work out that $\bar{x}_\Delta \mid \mu_\Delta$ has a t distribution, as

$$f(\bar{x}_\Delta \mid \mu_\Delta) = \int \mathcal{L}(\bar{x}_\Delta \mid \mu_\Delta, \sigma_0^2) g(\sigma_0^2) \mathrm{d}\sigma_0^2 \propto \left[1 + \frac{1}{c} \cdot \frac{(\bar{x}_\Delta - \mu_\Delta)^2}{\left(\frac{1}{n_A}+\frac{1}{n_B}\right)\sum p_k^2\xi_k^2}\right]^{-\frac{c+1}{2}}.$$

Likewise, we relate it with the normal kernel conditional on unknown σ_0^2 so that

$$f(\bar{x}_\Delta \mid \mu_\Delta, \sigma_0^2) \propto \frac{1}{\sigma_0} \exp\left(\frac{(\bar{x}_\Delta - \mu_\Delta)^2}{2\left(\frac{1}{n_A}+\frac{1}{n_B}\right)\sigma_0^2}\right).$$

Placing the normal prior distribution for μ_Δ, specified based on pre-experimental data, we obtain the distribution for $\bar{x}_\Delta$ unconditional on μ_Δ as

$$\bar{x}_\Delta \mid \sigma_0^2, \boldsymbol{y}_1, \ldots, \boldsymbol{y}_K \sim N\left(\sum p_k\lambda_k, \left(\frac{1}{n_A}+\frac{1}{n_B}\right)\sigma_0^2 + \sum_k p_k^2\xi_k^2\right),$$

which depends on the distribution for σ_0^2.

F. KEY PARAMETERS ALTERING THE INFORMATIVENESS OF THE COLLECTIVE PRIOR

In our Bayesian modelling, the prior probabilities of incommensurability, w_k, $k = 1, \ldots, K$, can attenuate the strength of individual commensurate predictive priors in a direct manner. To form a collective prior, we obtain the weights, $p_1, \ldots, p_K$, using a non-decreasing function of w_k, which was shown to have desired properties by Zheng and Wason (2022) [1]. As noted, a value of $s_0 \to 0^+$ can discern the levels of pairwise (in)commensurability between pre-trial and the new parameters more sensitively, so the collective prior, $N(\sum p_k\lambda_k, \sum p_k^2\xi_k^2)$, would be dominated by the pre-experimental information (down-weighted to an appropriate level via w_k) thought of as the most consistent.

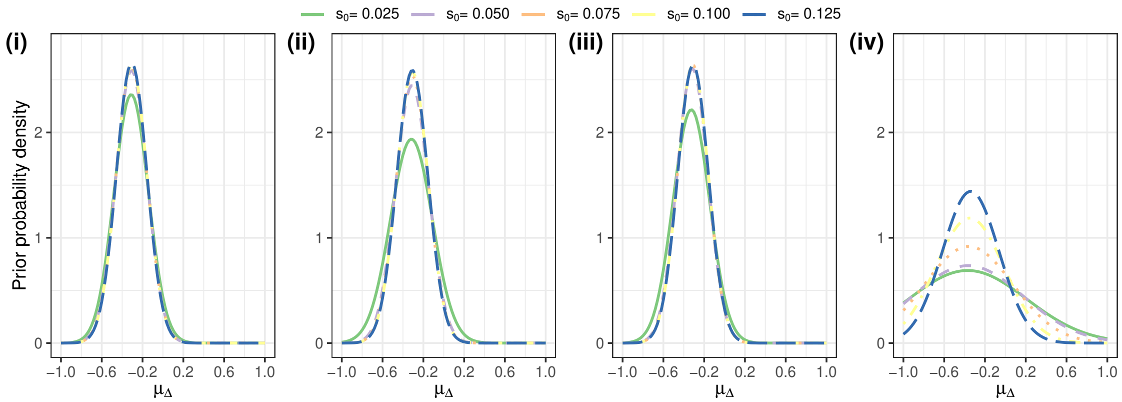

Figure S2: Possible collective priors formed based on the five sets of consistent pre-experimental information, for which different levels of down-weighting could have been accounted.

Figure S2 shows how these key parameters may alter the informativeness of the collective prior for the five sets of expert opinion in Section 3 of the main manuscript, which was supposed to have been summarised as $N(-0.26, 0.25)$, $N(-0.24, 0.23)$, $N(-0.37, 0.22)$, $N(-0.34, 0.36)$ and $N(-0.32, 0.26)$. In particular, the subfigures (i) – (iv) are from the following specifications of w_k, respectively:

- $w_1 = 0.15$, $w_2 = 0.20$, $w_3 = 0.17$, $w_4 = 0.13$, $w_5 = 0.20$ (used in the Application section);
- $w_1 = 0.20$, $w_2 = 0.20$, $w_3 = 0.20$, $w_4 = 0.10$, $w_5 = 0.20$;
- $w_1 = 0.20$, $w_2 = 0.20$, $w_3 = 0.10$, $w_4 = 0.20$, $w_5 = 0.20$;
- $w_1 = 0.50$, $w_2 = 0.50$, $w_3 = 0.10$, $w_4 = 0.50$, $w_5 = 0.50$.

Referring to subfigure (i), where $w_1, \ldots, w_K$ are prespecified to be comparable fractions for such consistent pre-experimental information, the tuning parameter s_0 has limited impact on altering the shape of the collective prior. Looking across subfigures (ii) and (iii), a smaller value chosen for s_0 drags the collective prior further down to the pre-experimental information thought of as more consistent (so a smaller w_k to obtain the corresponding commensurate predictive prior). From subfigure (iii) to (iv), for which pre-experimental information is discounted to an even larger extent, the informativeness of the collective prior consequently diminishes. In addition, subfigure (iv), when compared with the rest in Figure S2, has $w_1, \ldots, w_K$ that are more distinctive between themselves; so setting $s_0 = 0.05$ picks up the least down-weighted pre-experimental information almost as sensitive as $s_0 = 0.025$.

We inspect the property with another set of pre-experimental information, summarised as $N(-0.26, 0.25)$, $N(-0.17, 0.15)$, $N(-0.44, 0.40)$, $N(-0.15, 1.89)$ and $N(0.12, 0.22)$, which has higher variability across available sources. We consider

- $w_1 = 0.30$, $w_2 = 0.10$, $w_3 = 0.30$, $w_4 = 0.30$, $w_5 = 0.30$;
- $w_1 = 0.30$, $w_2 = 0.30$, $w_3 = 0.30$, $w_4 = 0.10$, $w_5 = 0.30$.

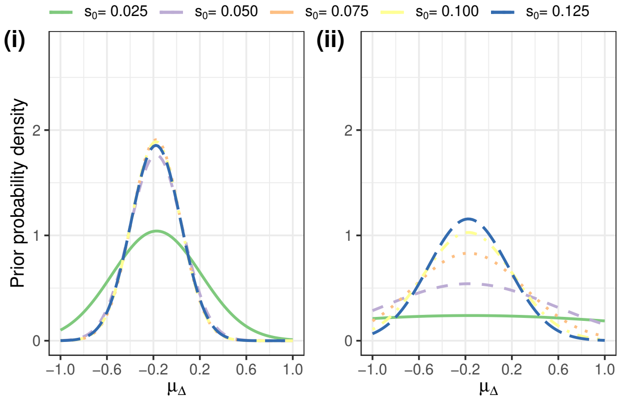

Figure S3: Possible collective priors formed based on divergent pre-experimental information.

Figure S3 displays the probability density function of a collective prior that could be, following our parameters specification. As expected, from subfigure (i) to (ii), the collective prior becomes less informative, since the extreme $N(-0.15, 1.89)$ summary is discounted to a smaller extent. It reaches to the same conclusion as above on the influence of s_0.

Based on all plots in this section, we recommend choosing a small (relative to the magnitude of $w_1, \ldots, w_K$) value for s_0, particularly when a heavy-tailed collective prior is desired.

G. PAIRWISE COMMENSURABILITY OF THE HYPOTHETICAL HISTORICAL DATA

We compute the squared Hellinger distance [2] for all pairs of the individually specified prior opinion on the log-odds ratio, $\theta_k \mid \boldsymbol{y}_k$, with each represented in a $N(m_k, s_k^2)$ distribution. The squared Hellinger distance between two normal distributions, say, $N(m_k, s_k^2)$ and $N(m_{k'}, s_{k'}^2)$, has a closed-form expression:

$$H^2(\theta_k \mid \boldsymbol{y}_k, \theta_{k'} \mid \boldsymbol{y}_{k'}) = 1 - \sqrt{\frac{2 s_k^2 s_{k'}^2}{s_k^2 + s_{k'}^2}} \exp\left(-\frac{(m_k - m_{k'})^2}{4(s_k^2 + s_{k'}^2)} \right).$$

Figure S4 visualised the pairwise Hellinger distances, i.e., the square root of the expression above, for the hypothetical pre-experimental data. This could be useful to choose the prior probabilities of incommensurability, w_k, $k = 1, \ldots, K$, for applying the proposed Bayesian sample size formulae.

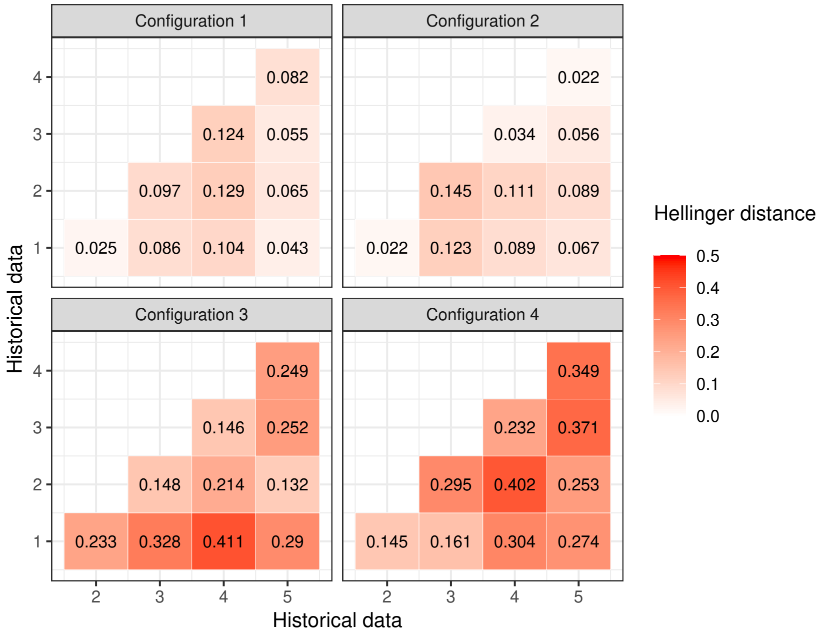

Figure S4: Pairwise distributional discrepancy between any two $\theta_k \mid \boldsymbol{y}_k \sim N(m_k, s_k^2)$.

H. SIMULATIONS TO EVALUATE THE AVERAGE PROPERTIES OF THE POSTERIOR

In this section, we simulate the continuous measurements X_{ij}, $j = A, B$, supposing that the new experiment would draw random samples of size n_A and n_B for comparison. These sample sizes are computed based on our proposed methodology. Recall that the allocation ratio, n_A/n_B, needs to be fixed for the computation. Without loss of generality, this simulation study investigates the case of equal allocation. As noted in the main paper, the entire probability space for sampling the new experimental data can be conceptualised by the marginal distribution of the difference in means, given pre-experimental information. More explicitly, we simulate X_{iA} from a $N\left(\sum_k p_k \lambda_k, \left(\frac{1}{n_A} + \frac{1}{n_B}\right) \sigma_0^2 + \sum_k p_k^2 \xi_k^2\right)$ distribution and X_{iB} from a $N\left(0, \left(\frac{1}{n_A} + \frac{1}{n_B}\right) \sigma_0^2 + \sum_k p_k^2 \xi_k^2\right)$ distribution, setting $\sigma_0^2 = 0.35$ for the true parameter for simulation. The posterior distribution will be obtained for cases of known and unknown variances, respectively.

Table S1: Operating characteristics of the new experiment, of which the sample size is based on our methodology. The target level of average coverage probability, interval length and expected variance are 0.95, 0.65 and 0.03, respectively.

			Evaluation metrics		
			Average coverage	Average interval length	Average variance
Known σ_0^2	Configuration 1	Robust weights I	0.945	0.650	0.030
		Robust weights II	0.947	0.647	0.030
	Configuration 2	Robust weights I	0.940	0.650	0.030
		Robust weights II	0.952	0.644	0.030
	Configuration 3	Robust weights I	0.951	0.650	0.030
		Robust weights II	0.947	0.646	0.030
	Configuration 4	Robust weights I	0.947	0.649	0.030
		Robust weights II	0.946	0.616	0.030
Unknown σ_0^2	Configuration 1	Robust weights I	0.977	0.705	0.021
		Robust weights II	0.994	0.674	0.015
	Configuration 2	Robust weights I	0.907	0.668	0.035
		Robust weights II	0.974	0.684	0.025
	Configuration 3	Robust weights I	0.987	0.712	0.019
		Robust weights II	0.995	0.716	0.015
	Configuration 4	Robust weights I	0.977	0.705	0.022
		Robust weights II	0.994	0.674	0.015

We consider the same configurations of historical data listed in Table 1 of the main paper. The corresponding sample sizes have been visualised in Figure 1, for both cases of known ($\sigma_0^2 = 0.35$) and unknown variance (setting $c = 3$). For each scenario, we simulate 100,000 replicates of the new experiment to investigate the (i) coverage of an interval of fixed length $\ell_0 = 0.65$ for experiments with an ACC sample size, (ii) credible interval length of a region giving 95% coverage for experiments with an ALC sample size, and (iii) the posterior variance for experiments with an APVC sample size. More precisely, the coverage is the probability that the true difference in means is contained within the interval $[\eta - \ell_0/2, \eta + \ell_0/2]$, where η is the posterior mean. The credible interval length is defined by expression (7) of the main paper.

Table S1 shows that the average properties of the posterior distribution can be maintained for the new experiment which would have been designed using the proposed Bayesian sample size formulae. Slightly more uncertainty is observed for the case of unknown variance under the same configuration of historical data, due to the inclusion of a minimally informative prior on σ_0^2 by setting $c = 3$. It is hence possible for the marginal posterior distribution for μ_Δ to be less informative than that for the case of known variance. This explains the results in Table S1, where the posterior under for the case of unknown variance, yields higher coverage probability yet wider credible interval length than that of known variance.

I. SENSITIVITY TO NON-NORMAL DATA

The utility of our Bayesian methodology is not limited to normally distributed data, but can be applicable to data sampled from asymmetric or heavy-tailed distributions. This generality stems from our formulation of the data likelihood, which is in terms of $\bar{X}_\Delta$, the difference in sample means, rather than the outcome measurements X_{ij} themselves, for $i = 1, \ldots, n_j$, $j = A, B$. By the central limit theorem , even if the outcome measurements are not normally distributed on the

original scale, the sample mean has an asymptotic distribution $\bar{X}_j \sim N(\mu_j, \sigma_0^2/n_j)$, for $j = A, B$, and further $\bar{X}_\Delta \sim N(\mu_\Delta, \sigma_0^2/n_A + \sigma_0^2/n_B)$. With a normal prior (based on pre-experimental data that are likewise not necessarily normal) placed on μ_Δ, the normality of the posterior, say, $f_p(\mu_\Delta \mid \bar{x}_\Delta)$, is assured even for the case of small sample sizes.

To confirm this point, we extend the simulation study to cover asymmetric data. Specifically, the asymmetric data are simulated from skewed normal distributions with a coefficient γ, which is the third standardised moment of the posterior distribution, to indicate the skewness. A $\gamma < 0$ ($\gamma > 0$) indicates negative (positive) skewness that the longer tail is to the left (right). By contrast, $\gamma = 0$ represents the normal distribution. We then average across the 100,000 replicates per scenario to compute the coverage probability, interval length and variance of the posterior distribution, i.e., $f_p(\mu_\Delta \mid \boldsymbol{y}_1, \ldots, \boldsymbol{y}_K, \boldsymbol{y}_{K+1})$, following the respective decision criteria.

Table S2: Average coverage probability, interval length and expected variance of the posterior, yielded by the proposed methodology for asymmetric data.

		Target level	Normal	Asymmetric (skewed normal)						
				$\gamma = -0.99$	$\gamma = -0.7$	$\gamma = -0.5$	$\gamma = 0$	$\gamma = 0.5$	$\gamma = 0.7$	$\gamma = 0.99$
Known σ_0^2	ACC	$1 - \alpha_0 = 0.95$	0.94591	0.94571	0.94520	0.94558	0.94582	0.94513	0.94537	0.94494
	ALC	$\ell_0 = 0.65$	0.65078	0.65110	0.65074	0.65056	0.64962	0.64921	0.64923	0.64941
	APVC	$\epsilon_0 = 0.03$	0.02947	0.02772	0.02772	0.02772	0.02772	0.02772	0.02772	0.02772
Unknown σ_0^2	ACC	$1 - \alpha_0 = 0.95$	0.94387	0.94407	0.94514	0.94456	0.94427	0.94346	0.94330	0.94415
	ALC	$\ell_0 = 0.65$	0.68058	0.68085	0.68025	0.67997	0.67861	0.67818	0.67825	0.67860
	APVC	$\epsilon_0 = 0.03$	0.02085	0.02077	0.02077	0.02077	0.02077	0.02077	0.02077	0.02077

Table S2 lists the numerical performance of our Bayesian sample size formulae for the design of a new experiment that compares two groups of data sampled from asymmetric distributions. We have concentrated on Configuration 1 to report these results, since the conclusion does not change for other scenarios. These results assures that our methodology can be applied more widely for comparing the difference between two groups.

J. AVERAGE PROPERTIES OF THE POSTERIOR FROM THE ORIGINAL MIXTURE PRIORS

Our methodology involves a normal approximation to the mixture of t distributions, for deriving a prior distribution that leverages pre-experimental information from multiple sources. We realise the possibility that our Bayesian methodology may be used for the design (i.e., for the sample size consideration) only, while the analysis of experimental data could be conducted using the original priors; specifically, the t mixture prior $f(\tilde{\theta}_k \mid \theta_k)$, yielded by the Gamma mixture prior on each ν_k, would not be approximated by a normal distribution in the form of expression (4) of the main paper. This section is thus focused on the evaluation of our methodology in a simulation study, where the design and analysis priors differ. We use the same configurations of historical data and prior specification as those in Section H above.

A total of 100,000 replicates of the new experiment have been simulated for each scenario, with the sample sizes n_A and n_B computed based on our closed-form formulae. We then fit the Bayesian model with the original Gamma mixture priors used for ν_k in the data analysis, using Markov chain Monte Carlo. The inference is based on a chain of 13,000 MCMC samples, sacrificing the first 3000 iterations as burn-in. Recall that the posterior distribution would again be a unimodal mixture distribution, as updated from the t mixture prior, $f(\tilde{\theta}_k \mid \theta_k)$. For simplicity, the coverage would likewise be the probability that the true difference in means is contained within the interval

Table S3: Operating characteristics of the new experiment, of which the sample size is based on our methodology, if analysed using original mixture priors without the normal approximation.

			Evaluation metrics	
			Average coverage	Average variance
Known σ_0^2	Configuration 1	Robust weights I	0.851	0.041
		Robust weights II	0.847	0.047
	Configuration 2	Robust weights I	0.859	0.037
		Robust weights II	0.853	0.045
	Configuration 3	Robust weights I	0.818	0.053
		Robust weights II	0.810	0.060
	Configuration 4	Robust weights I	0.824	0.049
		Robust weights II	0.799	0.056
Unknown σ_0^2	Configuration 1	Robust weights I	0.822	0.041
		Robust weights II	0.826	0.046
	Configuration 2	Robust weights I	0.826	0.038
		Robust weights II	0.840	0.043
	Configuration 3	Robust weights I	0.763	0.052
		Robust weights II	0.813	0.060
	Configuration 4	Robust weights I	0.770	0.047
		Robust weights II	0.707	0.056

$[\eta_{\text{tmix}} - \ell_0/2, \eta_{\text{tmix}} + \ell_0/2]$, where η_{tmix} is the mean of the posterior t mixture distribution. It is not obvious how to find the length of a highest density region that gives a definitive coverage probability for a mixture distribution. So we would concentrate the results interpretation on the average coverage and average variance of the posterior distribution.

As Table S3 shows, the average posterior variance is maintained at similar levels, whereas the average coverage probability of the posterior decreases from the target level by 10% or more. This is because this metric concerns the area of highest density region, stretching to be symmetric about the mode, given a fixed length of credible interval.

The unimodal t mixture distribution, if not replaced by the approximated normal distribution, is more spreaded to the tails. A decrease in the coverage would be expected when a heavier tailed distribution is used in the analysis, since it has less mass in the highest density region that centres the mean. Moreover, we note that the number of information sources ($K = 5$) should account for the 10% or more decrease. In other words, each normal approximation to a t mixture distribution, specific to source $k = 1, \ldots, K$, contributes to the decreased coverage; when K gets larger, the loss tends to be cumulative (depending on the configuration of historical data as well as the weights for synthesis).

In conclusion, the results in Sections H and J illustrate that our methodology maintains the average properties of the posterior as expected. In particular, the normal approximation, which gives great advantages for deriving the closed-form sample size formulae, does not jeopardise the precision of posterior inference under various sets of weights. We recommend that the user uses the same prior for both the design and analysis, since the ‘two-prior’ approach (using our normal approximated prior for the design but the unapproximated in the analysis) would lead to lower coverage probability.

K. SAMPLE SIZE DETERMINATION FOR EXPERIMENTS WITH TIME-TO-EVENT DATA

The proposed Bayesian methodology is useful in the trial design setting of comparing the survival or hazard of two groups. We assume that the event times follow an exponential distribution [3]. That is, the event times $T_{ij} \sim \text{Exp}(\pi_j)$ for patient $i = 1, \ldots, n_j$ assigned to treatment group $j = A, B$. Let $\bar{T}_j$ be the average event time over patients in the same group j. By the central limit theorem,

$$\bar{T}_j \dot{\sim} N\left(\frac{1}{\pi_j}, \frac{1}{d_j \pi_j}\right),$$

where d_j is the number of events per group $j = A, B$. Applying the delta method, we obtain

$$\log(\bar{T}_j) \dot{\sim} N\left(-\log(\pi_j), \frac{1}{d_j}\right).$$

Due to the independence of data from the two treatment groups, it further leads to

$$\log\left(\frac{\bar{T}_A}{\bar{T}_B}\right) \dot{\sim} N\left(\log(\pi_\Delta), \frac{1}{d_A} + \frac{1}{d_B}\right).$$

Here, $\pi_\Delta = \pi_B/\pi_A$ is the hazard ratio, more precisely, the ratio of mean event times. As an alternative, one may consider modelling the ratio of median event times.

We suppose that there are K relevant pre-experimental datasets, and each can be summarised in a normal predictive prior, that is, $N(m_k, s_k^2)$, $k = 1, \ldots, K$, for $\log(\pi_\Delta)$. Let $w_1, \ldots, w_K$ denote the prior probabilities of incommensurability, respectively. Following our methodology, elaborated upon Section 2 of the main paper, a collective prior can thus be given by

$$\log(\pi_\Delta) \mid \boldsymbol{y}_1, \ldots, \boldsymbol{y}_K \sim N(\sum_k p_k \lambda_k, \sum_k p_k^2 \xi_k^2),$$

where the synthesis weights $p_1, \ldots, p_K$ can be transformed from the values of $w_1, \ldots, w_K$, $\lambda_k = m_k$ and $\xi_k^2 = s_k^2 + \frac{w_k b_{01}}{a_{01}-1} + \frac{(1-w_k) b_{02}}{a_{02}-1}$. Pragmatically, s_k^2 could be stipulated as $\frac{1}{d_{Ak}} + \frac{1}{d_{Bk}}$ where d_{jk} denotes the number of events of a historical trial k, if all historical trials have the same data structure as the new trial under planning.

Using Bayes' Theorem, one can obtain a collective posterior that

$$\log(\pi_\Delta) \mid \boldsymbol{y}_1, \ldots, \boldsymbol{y}_K, \boldsymbol{y}_{K+1} \sim N\left(\eta, \left(\frac{1}{\sum p_k^2 \xi_k^2} + \frac{1}{\frac{1}{d_A} + \frac{1}{d_B}}\right)^{-1}\right), \tag{S8}$$

where

$$\eta = \frac{\frac{1}{d_A} + \frac{1}{d_B}}{\sum p_k^2 \xi_k^2 + \frac{1}{d_A} + \frac{1}{d_B}} \sum p_k \lambda_k + \frac{\sum p_k^2 \xi_k^2}{\sum p_k^2 \xi_k^2 + \frac{1}{d_A} + \frac{1}{d_B}} \log(\bar{t}_A/\bar{t}_B).$$

The sample size formula based on the ACC can be derived as

$$\frac{d_A d_B}{d_A + d_B} \geq \frac{4 z_{\alpha/2}^2}{\ell_0^2} - \frac{1}{\sum p_k^2 \xi_k^2} \tag{S9}$$

where $z_{\alpha/2}$ is the upper $(\alpha/2)$-th quantile of the standard normal distribution, i.e., $\Phi^{-1}(1-\alpha/2)$, and ℓ_0 is the length of the HPD interval. The same sample size would be required if applying the

ALC, since the posterior variance does not involve an unknown parameter [4]. According to the APVC, the sample size formula becomes

$$\frac{d_A d_B}{d_A + d_B} \geq \frac{1}{\epsilon_0} - \frac{1}{\sum p_k^2 \xi_k^2} \tag{S10}$$

where ϵ_0 is the level of desired dispersion.

Finally, we recognise that the derivation of new (event-driven) sample size formulae for time-to-event data has simplified the censoring assumptions. Further work would be needed to deal with complexity caused by censoring, accrual rate and so forth.

L. IMPLEMENTING THE SAMPLE SIZE FORMULAE IN PRACTICE

The proposed sample size formulae involve a number of parameters to specify or elicit. Some of these can be estimated based on pre-experimental information. Table S4 gives a step-by-step guide to setting the parameters for the use of this methodology in practice.

Table S4: A brief user-guide for the design and analysis of a new experiment, for which historical data or expert opinion could be leveraged.

Step	Action	Parameter(s) to specify	Note	Software*
(1)	Choose a sensible SSD criterion, say, ACC, ALC or APVC.	α_0, or ℓ_0, or ϵ_0	Targeting a smaller α_0, ℓ_0 or ϵ_0 would mean a larger sample size is needed. There is no convention for setting these thresholds. They are likely to vary case by case.<br>The statistician may follow Figure 3 of the main paper to generate one for their case. This could facilitate the communication with the stakeholder, so seek a sample size that is both affordable and sensible to compare A and B.	The **Bayesian SSD using robust commensurate priors.R** script contains R functions, such as ACCknvar(R, sig02, alpha = 0.05, l0, wk, sk2, dw, br, s0), for the sample size determination.<br>**Figures1-4.R** has command lines to reproduce Figure 3.
(2)	Identify relevant pre-experimental data to inform the specification of a prior for μ_Δ. If there are none, elicition of expert opinion on the magnitude of μ_Δ would be needed.	$s_1^2, \ldots, s_K^2$	These are the associated levels of uncertainty inherent to historical data $k = 1, \ldots, K$. Each set of historical samples are analysed to obtain $N(m_k, s_k^2)$ distributions to describe the difference in means, or the log-odds ratio (for binomial data). For instance, m_k and s_k^2 can be the mean and variance of the respective historical posterior for $\theta_k \mid \boldsymbol{y}_k$.<br>In situations with no historical studies, the statisticians may come up with questions to elicit expert opinion about the magnitude of those quantities following, for example, the SHELF framework. [5]	The second column of, e.g., MySc1, in **Historical data.R** corresponds to s_k^2, for $k = 1, \ldots, K$.

Continued.

Table S4 – *Continued.*

Step	Action	Parameter(s) to specify	Note	Software*
(3)	Enable borrowing of information.	$w_1, \ldots, w_K$; $p_1, \ldots, p_K$	The users are advised to compute the pairwise Hellinger distances between the $N(m_k, s_k^2)$ distributions, for $k = 1, \ldots, K$, which represent the K sets of pre-experimental information; see Section G with Figure S4 in this document. Then, $p_1, \ldots, p_K$, can be transformed from $w_1, \ldots, w_K$ following a decreasing function such as $$p_k = \frac{\exp(-w_k^2/s_0)}{\sum_k \exp(-w_k^2/s_0)},$$ where a small value (relative to w_k) for s_0 is recommended. The user may find Section F with Figures S2 and S3 useful to set s_0. Pragmatically, an internal pilot approach may be considered to gather information for setting $w_1, \ldots, w_K$.	The R function, HdMat(mu.hist, var.hist), contained in **Bayesian SSD using robust commensurate priors.R** can be used to compute $w_1, \ldots, w_K$ from historical data based statistics, and pq(w0, s0) used to transform $p_1, \ldots, p_K$ from $w_1, \ldots, w_K$.
(4)	Understand the level of data variability in the new experiment.	σ_0^2 or c	In clinical trials where pilot studies have been conducted, choosing a value of σ_0^2 can be straightforward. If no data are available to set σ_0^2, borrowing of information to inform the unknown variance is possible: setting c to a large (small) value means substantial (limited) borrowing. The statistician may visualise the change of sample size with the increase of c, following the pattern of our Figure 2 in the main paper for the user's case.	**Figures1-4.R** has command lines to reproduce Figure 2.
(5)	Specify the Gamma mixture priors.	$a_{01}, b_{01}, a_{02}, b_{02}$	The only constraint is $a_{01}, a_{02} > 1$. A useful default setting is $a_{01} = b_{01} = 2$, $a_{02} = 54$, $b_{02} = 3$, which accommodates very limited borrowing and pooling as the two extremes.	The corresponding arguments are dw = c(a_{01}, b_{01}), br = c(a_{02}, b_{02}) in any R function for the derived sample size formulae in **Bayesian SSD using robust commensurate priors.R**
(6)	Run the new experiment, and collect data to analyse.	-	Same values specified in Steps (1) – (5) should be used in the Bayesian analysis to derive a posterior for μ_Δ.	
(7)	Conclude the comparison based on the posterior, $f_p(\mu_\Delta \mid \boldsymbol{y}_1, \ldots, \boldsymbol{y}_K, \boldsymbol{y}_{K+1})$.	-	If the sample size has been calculated based on the ACC (or ALC), the coverage (or the credible interval length) should be reported accordingly.	

*Software for statistical computing is made available at https://github.com/haiyanzheng/SSDcmspriors.

References

1. Zheng, H. and Wason, J. M. S. (2022). Borrowing of information across patient subgroups in a basket trial based on distributional discrepancy. *Biostatistics*, **23**(1), 120 – 135.

2. Dey, D. K. and Birmiwal, L. R. (1994). Robust Bayesian analysis using divergence measures. *Statistics & Probability Letters* **20**(4), 287 – 294.

3. George, S. L. and Desu, M. M. (1974). Planning the size and duration of a clinical trial studying the time to some critical event. *Journal of Chronic Diseases* **27**(1), 15 – 24.

4. Joseph, L. and Bélisle, P. (1997). Bayesian sample size determination for normal means and differences between normal means. *Journal of the Royal Statistical Society: Series D (The Statistician)* **46**(2), 209 – 226.

5. Oakley, J. E. and O'Hagan, A. (2010). SHELF: the Sheffield elicitation framework (version 4.0), Sheffield, UK: School of Mathematics and Statistics, University of Sheffield.
